# Muscle stem cells and fibro-adipogenic progenitors in female pelvic floor muscle regeneration following birth injury

**DOI:** 10.1038/s41536-022-00264-1

**Published:** 2022-12-16

**Authors:** Francesca Boscolo Sesillo, Varsha Rajesh, Michelle Wong, Pamela Duran, John B. Rudell, Courtney P. Rundio, Brittni B. Baynes, Louise C. Laurent, Alessandra Sacco, Karen L. Christman, Marianna Alperin

**Affiliations:** 1 Department of Obstetrics, Gynecology, and Reproductive Sciences, Division of Female Pelvic Medicine and Reconstructive Surgery, University of California, San Diego, San Diego, CA 92037 USA; 2 Sanford Consortium for Regenerative Medicine, La Jolla, CA 92037 USA; 3 Division of Biological Sciences, University of California, San Diego, La Jolla, CA 92161 USA; 4 Department of Bioengineering, University of California, San Diego, La Jolla, CA 92093 USA; 5 Department of Obstetrics, Gynecology, and Reproductive Sciences, Division of Maternal-Fetal Medicine, University of San Diego, La Jolla, CA 92037 USA; 6 Development, Aging and Regeneration Program, Sanford Burnham Prebys Medical Discovery Institute, La Jolla, CA 92037 USA

**Keywords:** Muscle stem cells, Stem-cell research

## Abstract

Pelvic floor muscle (PFM) injury during childbirth is a key risk factor for pelvic floor disorders that affect millions of women worldwide. Muscle stem cells (MuSCs), supported by the fibro-adipogenic progenitors (FAPs) and immune cells, are indispensable for the regeneration of injured appendicular skeletal muscles. However, almost nothing is known about their role in PFM regeneration following birth injury. To elucidate the role of MuSCs, FAPs, and immune infiltrate in this context, we used radiation to perturb cell function and followed PFM recovery in a validated simulated birth injury (SBI) rat model. Non-irradiated and irradiated rats were euthanized at 3,7,10, and 28 days post-SBI (dpi). Twenty-eight dpi, PFM fiber cross-sectional area (CSA) was significantly lower and the extracellular space occupied by immune infiltrate was larger in irradiated relative to nonirradiated injured animals. Following SBI in non-irradiated animals, MuSCs and FAPs expanded significantly at 7 and 3 dpi, respectively; this expansion did not occur in irradiated animals at the same time points. At 7 and 10 dpi, we observed persistent immune response in PFMs subjected to irradiation compared to non-irradiated injured PFMs. CSA of newly regenerated fibers was also significantly smaller following SBI in irradiated compared to non-irradiated injured PFMs. Our results demonstrate that the loss of function and decreased expansion of MuSCs and FAPs after birth injury lead to impaired PFM recovery. These findings form the basis for further studies focused on the identification of novel therapeutic targets to counteract postpartum PFM dysfunction and the associated pelvic floor disorders.

## Introduction

Female pelvic floor muscles (PFMs) span pelvic outlet to support pelvic and abdominal viscera, aid in urinary and fecal continence, and enable sexual function. In humans, PFMs include the levator ani complex, comprised of the medial (puborectalis) and lateral (pubococcygeus) portions of pubovisceralis and iliococcygeus, and the posterior coccygeus muscle^1^. PFM dysfunction is one of the leading contributors to the development of pelvic floor disorders that include pelvic organ prolapse, and urinary and fecal incontinence^2^. These morbid and costly conditions negatively impact quality of life of close to a quarter of the U.S. female population^2^. Maternal trauma to PFMs during vaginal delivery due to eccentric contractions (natural childbirth) or passive stretch (childbirth under regional anesthesia) is the key risk factor for PFM dysfunction; however, not all women develop this condition consequent to childbirth^2^. It is possible that the disparity in PFM dysfunction consequent to parturition is, at least in part, due to the differential endogenous potential for PFM regeneration. Despite the dramatic prevalence of maternal PFM injury and a large number of women affected by the related morbid pelvic floor disorders, little research has been done to understand PFM regeneration following vaginal childbirth. The above served as the main impetus for the current study.

Skeletal muscle regeneration, mainly studied in the limb muscles, consists of a tightly orchestrated series of events that involve multiple cell types. Muscle stem cells (MuSCs) are muscle resident cells, which play a major role in the regenerative process^3,4^. During muscle regeneration, MuSCs, which are quiescent in homeostatic conditions, become activated, proliferate, and eventually differentiate and fuse with existing myofibers or form new ones^5^. The contributions of fibro-adipogenic progenitors (FAPs) and immune cells are essential to achieve efficient regeneration^6,7^. FAPs are muscle-resident mesenchymal cells located in the interstitial space between myofibers^7,8^. Similar to MuSCs, they start proliferating after muscle injury, supporting MuSCs activation and expansion through cytokine secretion^7,9^. Immune cells are recruited to the site of injury as early as 1 h after muscle damage occurs^10,11^. Neutrophils that are the “first responders” secrete pro-inflammatory cytokines, inducing the recruitment of monocytes and macrophages to the site of injury^10^. Macrophages first differentiate into pro-inflammatory (M1) and subsequently anti-inflammatory (M2) phenotypes in response to changes in the environmental cues and cytokine milieu^11^. Interactions among these different cell types are essential for proper muscle regeneration^9,12^.

The majority of the existing studies focused on cellular events during muscle regeneration are performed in murine hind limb muscles subjected to either myotoxic (cardiotoxin, notexin), chemical (barium chloride), denervation, or cryo-injury^13–15^. Moreover, these studies are conducted primarily in male animal models, leaving the female muscle regenerative biology highly understudied. Significant sexual dimorphisms exist in skeletal muscles. At the transcriptional level, numerous genes involved in the regulation of muscle mass are differentially expressed in male vs. female muscles, resulting in abundant differences, including fiber phenotype and contractility^16,17^. In addition, several studies demonstrate differences between sexes in myoblast potency in vitro or after transplantation. Male-derived myoblasts have increased proliferative ability when isolated from adult animals or cultured for more than 3 months, while transplanted female cells have better implantation potential^18,19^. However, when isolated MuSCs were transplanted, no differences in implantation were observed between male and female muscles^20^. Thus, studies focused on female muscle regeneration in general and on pelvic skeletal muscles specifically are needed to expand the existing literature and to help resolve these conflicting results. Furthermore, despite dramatic prevalence of pelvic floor disorders that disproportionately affect parous women, to our knowledge, there are no published studies evaluating the role of MuSCs, FAPs, or immune response in postpartum PFM regeneration.

Given the ethical and technical limitations associated with the use of human participants for the studies of PFMs, we utilized the rat model, previously validated for the investigations of human PFMs and the impact of birth injury on pelvic soft tissues^21–24^. The rat PFM complex is analogous to humans, consisting of the pubocaudalis (PCa) and iliocaudalis (ICa) muscles that together comprise rat levator ani, and the coccygeus (C) muscle^21^. Employing the well-established simulated birth injury (SBI) model, we aimed to assess the contribution of MuSCs, FAPs, and immune cells to PFM regeneration. We hypothesized that together with coordinated immune response, MuSCs and FAPs play a major role in female PFM constructive remodeling after birth injury. To explicitly test our hypothesis, we relied on irradiation to perturb cellular function.

## Results

### Pelvic floor muscle regeneration following simulated birth injury is impaired after whole muscle irradiation

**Fig. 1:**
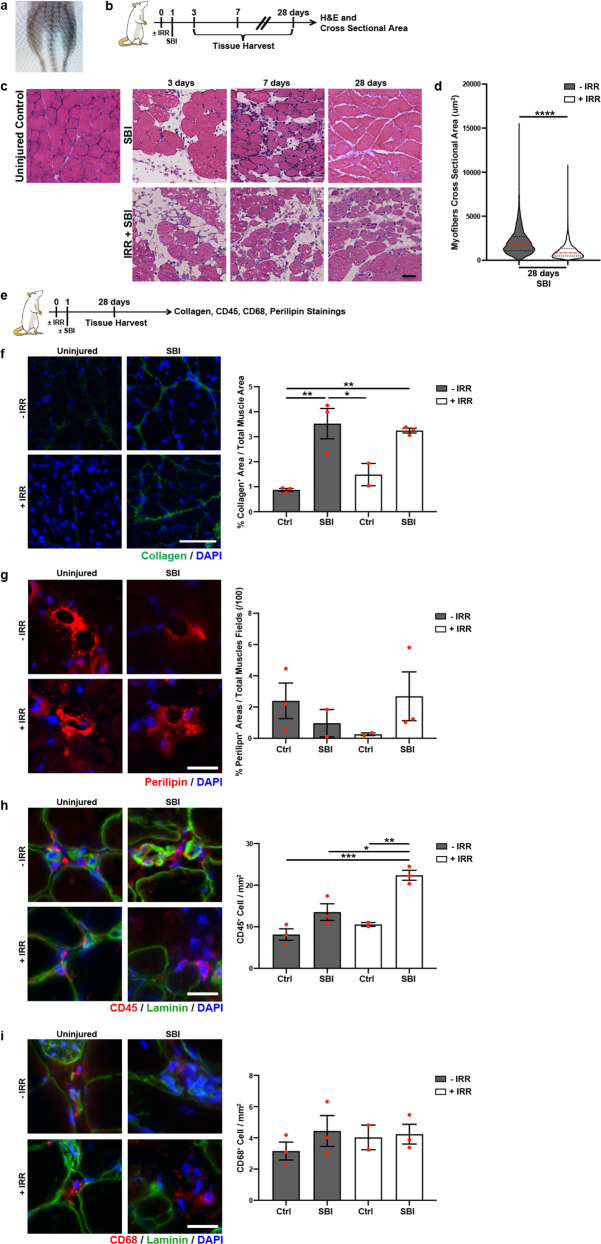
The impact of simulated birth injury on the morphometric properties of pubocaudalis (PCa) in nonirradiated and irradiated rat model. **a** X-ray image of the irradiated area. **b** Schematic representation of the experimental design for C. **c** H&E staining of PCa at 3, 7, and 10 days after injury (scale bar: 50μm). **d** Violin plots representing distribution of the myofiber size at 28-day time point. ****p value < 0.0001; Mann-Whitney test. *n* = 3 animals. **e** Schematic representation of the experimental design for F. **f** On the left, representative immunofluorescent images of collagen staining (scale bar: 100μm). On the right, quantification of the collagen area fraction. **g** On the left, representative immunofluorescent images of perilipin staining (scale bar: 25 µm). On the right, quantification of the perilipin^+^ cells per mm^2^. **h** On the left, representative immunofluorescent images of CD45 staining (scale bar: 25 µm). On the right, quantification of the CD45^+^ cells per mm^2^. **i** On the left, representative immunofluorescent images of CD68 staining (scale bar: 25 µm). On the right, quantification of the CD68^+^ cells per mm^2^. Red dots represent individual measurements; error bar represent SEM. *p value < 0.05; **p value < 0.01. One-way ANOVA with Tukey’s post hoc. *n* = 3 animals.

To assess the regenerative potential of PFMs and determine cellular events necessary for muscle recovery following SBI, we employed irradiation with a single 20 Gy dose (Fig. 1a), which is in the range shown to comprise MuSC function in limb muscles. Quiescent and low proliferating cells are less affected than highly proliferating cells, thus, to perturb these cells functionality, a higher irradiation dose is required^25^. Indeed, the MuSC function has been shown to be compromised when 18–25 Gy are applied to the muscle^26^. SBI was performed via vaginal balloon distention that replicates circumferential and downward strains imposed on PFMs during parturition^27^. First, we sought to determine the impact of stem and progenitor cellular impairment on muscle regeneration by comparing the histomorphology of PCa 3, 7, and 28 days postinjury in irradiated and non-irradiated animals (Fig. 1b, c). PCa was selected as a starting point because it sustains the largest strains during delivery compared to the other component of the rat levator ani complex, analogous to the pubovisceralis portion of the human levator ani muscle^22–24,28^. Our findings are consistent with the published data, with all PCa muscles demonstrating large injured areas, whereas only two-thirds of the ICa samples exhibited signs of muscle damage. Approximately 20% of the C muscles was spared from the impact of SBI. The extent of injury was also evaluated indirectly through the assessment of the embryonic myosin heavy chain (eMyHC) expression in the non-irradiated muscles 7 days post-SBI, and was found to be the highest in PCa, followed by C, and then ICa, consistent with the histomorphological muscle appearance (Supplementary Fig. 1a,b).

The extent of injury in PCa from the irradiated and non-irradiated groups was comparable, as evident from the large interstitial spaces 3 days post-SBI (Fig. 1c). As expected, regenerating myofibers, identified by centralized nuclei, were present in non-irradiated animals 7 days postinjury. On the other hand, animals irradiated before injury did not show signs of muscle regeneration at this time point (Fig. 1c). One month after injury, the differences between the two groups were even more pronounced. While PCa morphology in non-irradiated injured animals did not fully return to the unperturbed state, PCa in irradiated injured animals exhibited significantly smaller myofibers and persistent large interstitial spaces despite a 4-week recovery period, demonstrating substantial impairment in regeneration compared to the non-irradiated injured muscles (Fig. 1c, d). Taken together, the above indicates that irradiation effectively impairs PFMs’ regenerative ability and thus can be efficiently deployed as a tool to identify the major players in PFMs’ recovery following birth injury.

Overall, we found a low amount of central nucleation in the early stages of PFM regeneration (3 and 7 days) compared to such observations in regenerating hind limb muscles. Intrigued by this observation, we asked whether this could be due to the type of injury imposed on the muscle. To address this question, we induced BaCl_2_ injury in PCa and compared the amount of central nucleation during regeneration (3, 7 and 10 days) to that in PCa subjected to SBI (Supplementary Fig. 1c, d). The number of centralized nuclei was significantly greater after BaCl_2_ injection compared SBI at days 3 and 10 postmuscle injury. PCa central nucleation 7 days after injury was greater in the BaCl_2_ compared to the SBI group, but the difference did not reach statistical significance_._ This suggests that SBI and BaCl_2_ induce a differential regenerative response in PFMs.

To investigate potential causes accountable for the increased interstitial space and cellular infiltrate observed in the irradiated injured animals, we assessed PFMs for possible fibrotic (collagen^+^ area fraction) and fatty (perilipin^+^ area fraction) degeneration, as well as for persistence of immune (CD45^+^ and CD68^+^) cells (Fig. 1e). With respect to extracellular matrix (ECM), we observed significant increase in intramuscular collagen, a major constituent of the muscle ECM in non-irradiated injured PFMs compared to uninjured controls (Fig. 1f), consistent with the previous studies conducted in our lab^29^. We found that the total PCa collagen content was not altered by irradiation in uninjured muscles when compared to non-irradiated unperturbed controls (Fig. 1f). Irradiation prior to SBI did not further increase fibrotic degeneration of injured PCa relative to non-irradiated counterparts (Fig. 1f). With respect to fatty degeneration, we did not observe significant differences in the amount of perilipin between groups. However, a downward trend in the intramuscular fat content was observed in animals that underwent either SBI alone or irradiation without birth injury compared to uninjured controls (Fig. 1g). Interestingly, perilipin distribution differed substantially in injured irradiated animals compared to unperturbed controls. While small perilipin^+^ areas were present throughout the interstitium of the whole muscle in the injured irradiated animals, large perilipin^+^ areas localized in proximity to the nerves and blood vessels were observed in unperturbed controls.

We found that CD45^+^ cells, a marker of all differentiated hematopoietic cells except erythrocytes and plasma cells, were significantly increased in injured irradiated animals 28 days after injury compared to all other conditions, indicating persistence of immune infiltrate in PFMs irradiated prior to SBI (Fig. 1h). To further delve into identity of these immune cells, we performed immunostaining for CD68, a pan-macrophage marker. We did not observe significant differences in the amount of macrophages between groups (Fig. 1i). These results suggest that the increased interstitial spaces observed in irradiated injured animals are occupied by the immune infiltrate, which was either not cleared from the muscle or accumulated during the regenerative process.

### Irradiation impairs pelvic floor muscle stem cell proliferation and fibro-adipogenic progenitor expansion in response to simulated birth injury

**Fig. 2:**
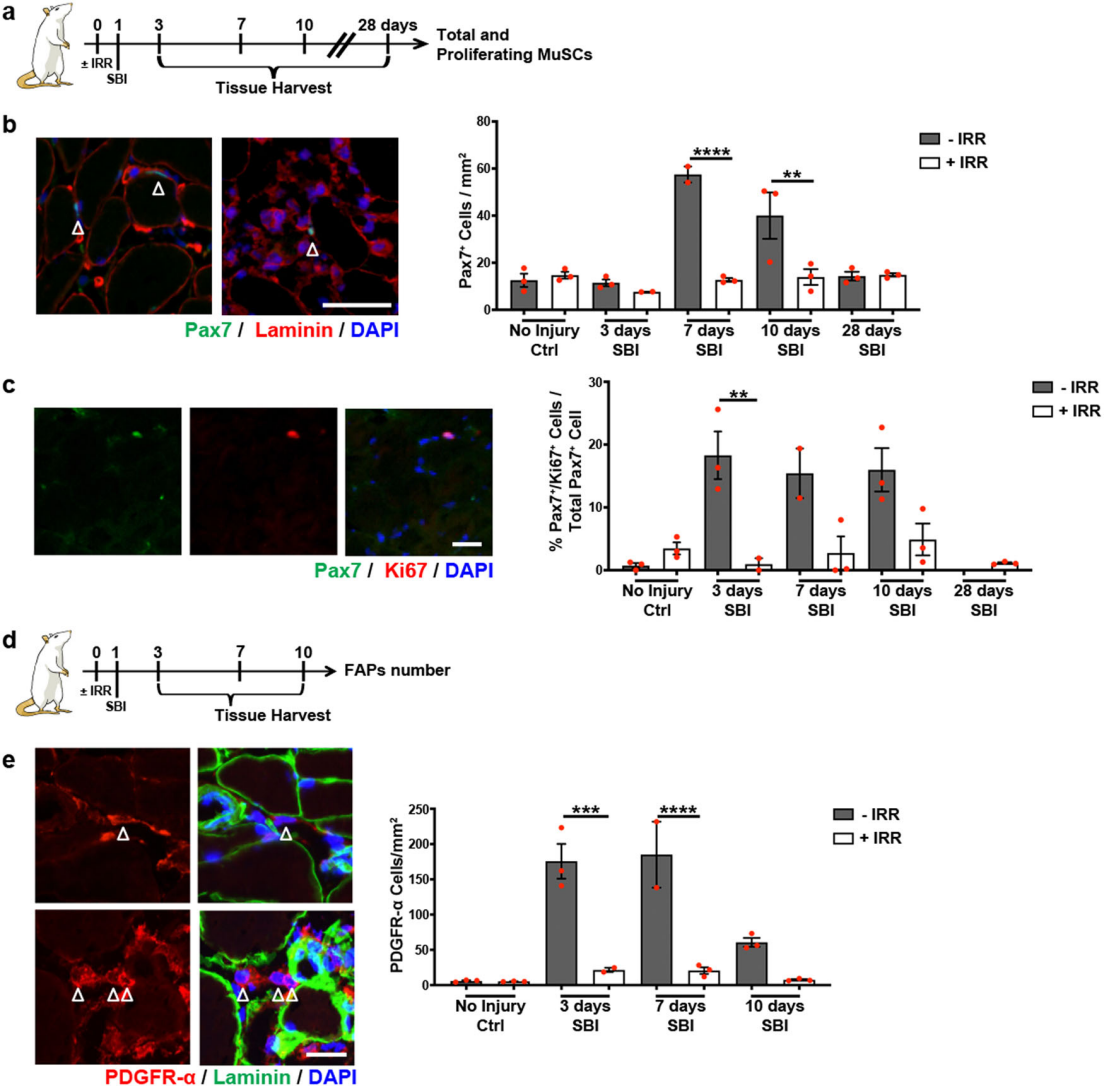
Muscle stem cells (MuSCs) and fibro-adipogenic progenitors (FAPs) behavior in regenerating pubocaudalis (PCa) muscle in non-irradiated and irradiated animals. **a** Schematic representation of the experimental design for B and C. **b**, **c**, and **d** Red dots represent single measurements; error bars represent SEM. **b** On the left, representative immunofluorescent images of Pax7/laminin staining of non-irradiated injured (left) and irradiated injured (right) PFMs 7 days following simulated birth injury. White arrows indicate MuSCs (scale bar: 50 μm). On the right, quantification of MuSC number per mm^2^. **p-value < 0.01; ****p-value < 0.0001; One-way ANOVA with Tukey’s post hoc. Complete Statistics in Supplementary Table 1. *n* = 3 animals. **c** On the left, representative immunofluorescent images of Pax7/Ki67 staining (scale bar: 25μm). On the right, quantification of Pax7/Ki67 double positive cells. *p-value < 0.05; **p-value < 0.01; One-way ANOVA with Tukey’s post hoc. *n* = 3 **d** Schematic representation of the experimental design for E. **e** On the left, representative immunofluorescent images of PDGFR-α/laminin staining. Top two images are of uninjured nonirradiated pubocaudalis; bottom two images are of non- irradiated pubocaudalis 3 days after simulated birth injury. White arrows indicate FAPs (scale bar: 25μm). On the right, quantification of FAP number per mm^2^. Red dots represent single measurements; error bar represent SEM. **p-value < 0.01; ***p-value < 0.001; ****p-value < 0.0001; One-way ANOVA with Tukey’s post hoc. *n* = 3 animals.

Irradiation is known to negatively impact the function of dividing cells, thus, we hypothesized that it would affect MuSC and FAP functionality following birth injury, leading to the long-term PFM phenotype described above. To explicitly test this hypothesis, we compared the response of PFM stem cells to birth injury in non-irradiated rats and animals irradiated 1 day before SBI (Fig. 2a, Supplementary Fig. 2a). As an initial step, we confirmed that the MuSC reservoir is not altered by irradiation in the otherwise unperturbed PFMs by employing Pax7 to identify and quantify MuSCs in situ. Pax7^+^ cell number did not differ 1 day after irradiation in any of the individual PFMs (PCa, ICa, C) compared to non-irradiated uninjured animals (Fig. 2b, Supplementary Figure 2b, c, Supplementary Table 1). The importance of the above is that at the time of SBI, the starting MuSC reservoir was not different between non-irradiated and irradiated animals.

We went on to assess the MuSC response to the birth injury. While the number of PCa MuSCs was similar in irradiated and non-irradiated animals 3 days after SBI (Fig. 2b), the significant increase in the number of MuSCs was observed at 7 and 10 days following birth injury in non-irradiated animals, consistent with our previous findings^29^. In contrast, this upsurge was not recapitulated in the irradiated group (Fig. 2b, Supplementary Table 1). The number of MuSCs returned to basal level 28 days after injury in the non-irradiated group (Fig. 2b, Supplementary Table 1). Similar to PCa results were observed for C, while MuSC number of ICa, which experiences the lowest strains during SBI^22^, did not differ significantly between non-irradiated and irradiated animals at any time point during the recovery period (Supplementary Fig. 2b, c). These findings suggest that irradiation perturbs MuSC activation and/or proliferation and that the impact of MuSCs loss is modulated by the magnitude of strain and related muscle injury.

To determine the potential cause of the quantitative differences in MuSCs following SBI between irradiated and non-irradiated groups, we went on to assess MuSC proliferation. Employing Ki67 as a marker of cell proliferation, we identified and quantified proliferating MuSCs in the regenerating PFMs. The number of proliferating MuSCs did not differ between irradiated and non-irradiated uninjured animals in any of the 3 individual PFMs (Fig. 2c, Supplementary Fig. 2d, e, Supplementary Table 2). However, consistent with the overall quantitative differences post-SBI, the proportion of proliferating MuSCs in PCa and C amplified only in the non-irradiated injured group, with no increase in MuSC proliferation observed in the irradiated injured animals. In contrast, no significant difference between groups was observed in ICa that sustains minimal injury. PFM stem cell proliferation returned to baseline at 28 days after SBI (Fig. 2c, Supplementary Fig. 2d, e, Supplementary Table 2). These results demonstrate that irradiation of PFMs negatively impacts MuSC proliferative ability during regeneration following birth injury, similarly to what has been observed in hind limb muscles^26^.

In addition to MuSCs, FAPs that aid in MuSC activation and proliferation are another important player in proper skeletal muscle regeneration. To our knowledge, direct proof of the negative effect of irradiation on FAP activation does not exist in the currently published literature. Thus, we first asked whether the function of these cells is affected by irradiation. Subsequently, we went on to determine the role of FAPs in PFM recovery following a birth injury. We performed immunohistochemistry using anti-PDGFR-α antibody to identify and quantify FAPs in uninjured and injured (3, 7, and 10 days post-SBI) PFMs of irradiated and non-irradiated animals (Fig. 2d). We did not observe any differences in the PCa FAP number in non-injured animals with or without irradiation (Fig. 2e, Supplementary Table 3). In response to injury, FAP number significantly increased in PCa of non-irradiated animals 3 and 7 days post-SBI, whereas this increase was not observed in irradiated injured animals at either time point (Fig. 2e, Supplementary Table 3). The FAP number was similar to the baseline level 10 days after injury (Fig. 2e, Supplementary Table 3). Findings in C were consistent with those in PCa; however, similar to the response of ICa MuSCs, the FAP number in this component of the PFM complex did not differ significantly between irradiated and non-irradiated injured animals (Supplementary Fig. 2f–h). Importantly, these results suggest that the ability of PFM FAPs to expand after SBI is impaired by irradiation. Overall, the lack of MuSC and FAPs expansion after birth injury directly correlates with the impairment in PFMs recovery, underscoring their role in the PFM regenerative process.

### PFMs’ neuromuscular junctions and blood vessels are not affected by irradiation

**Fig. 3:**
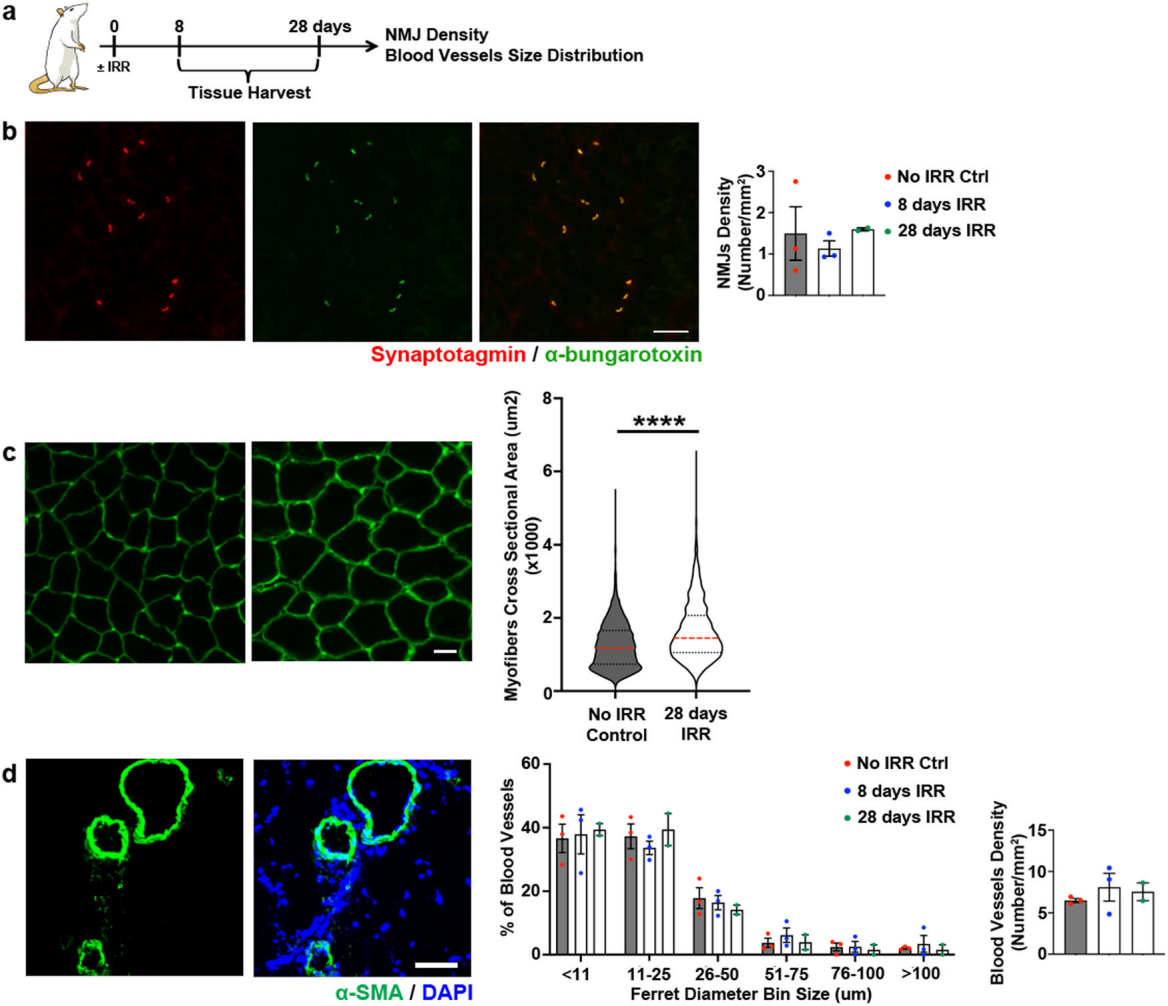
Neuromuscular junctions and blood vessels in irradiated uninjured animals. **a** Schematic representation of the experimental design for B and C. **b** On the left, representative immunofluorescent images of synaptotagmin (in red) andα-bungarotoxin (in green) staining (scale bar: 100 µm). On the right, bar graph representing NMJ density. **c** On the left, representative immunofluorescent images of laminin staining (scale bar: 100 µm). On the right, violin plot representing myofibers cross sectional area distribution of irradiated and non-irradiated PCa muscle at 28 days timepoint. ****p value < 0.0001; Mann-Whitney test. *n* = 3 animals. **d** On the left, representative immunofluorescent images of α-SMA staining (scale bar: 50 µm). In the center, bar graph representing the distribution of blood vessel ferret diameters. On the right, bar graph representing blood vessel density. Colored dots represent single measurements; error bar represent SEM. One-way ANOVA with Tukey’s post hoc. *n* = 3 animals.

Proper muscle regeneration requires efficient muscle innervation and temporally regulated immune response. Muscle denervation negatively affects muscle recovery postinjury through the reduction of the FAPs ability to aid MuSCs in the process of regeneration^30^. Thus, we assessed whether irradiation impacts neuro-muscular junctions (NMJ). At 8 and 28 days after irradiation, NMJ density in any of the three individual PFMs did not differ from the unperturbed muscles (Fig. 3a, b, Supplementary Fig. 3b, c). In addition, to determine whether NMJ were functional in irradiated animals we compared PCa myofiber cross-sectional area (CSA) between uninjured controls and animals 28 days postirradiation (Fig. 3c). This time point was selected based on the published data, showing major decrease in muscle CSA 30 days after denervation^30^. The decrease in fiber size was not observed in irradiated PCa compared to non-irradiated muscles, indicating that irradiation did not lead to the loss of NMJ functionality in our model (Fig. 3c).

Recruitment of circulating leukocytes from vasculature to the site of injury is necessary to clear the debris, which, in turn, aids in the activation and differentiation of MuSCs and FAPs^12^. To determine whether irradiation affects intramuscular blood vessels, potentially impairing the access of immune cells to PFMs, we examined arterioles at 8 and 28 days after irradiation using an antibody against α-smooth muscle actin (α-SMA), a smooth muscle cell marker (Fig. 3a, Supplementary Fig. 3a). Irradiation alone did not affect arteriolar size or density at either time point (Fig. 3d, Supplementary Fig. 3f, g).

### Immune response of the pelvic floor muscles to birth injury is increased by irradiation

**Fig. 4:**
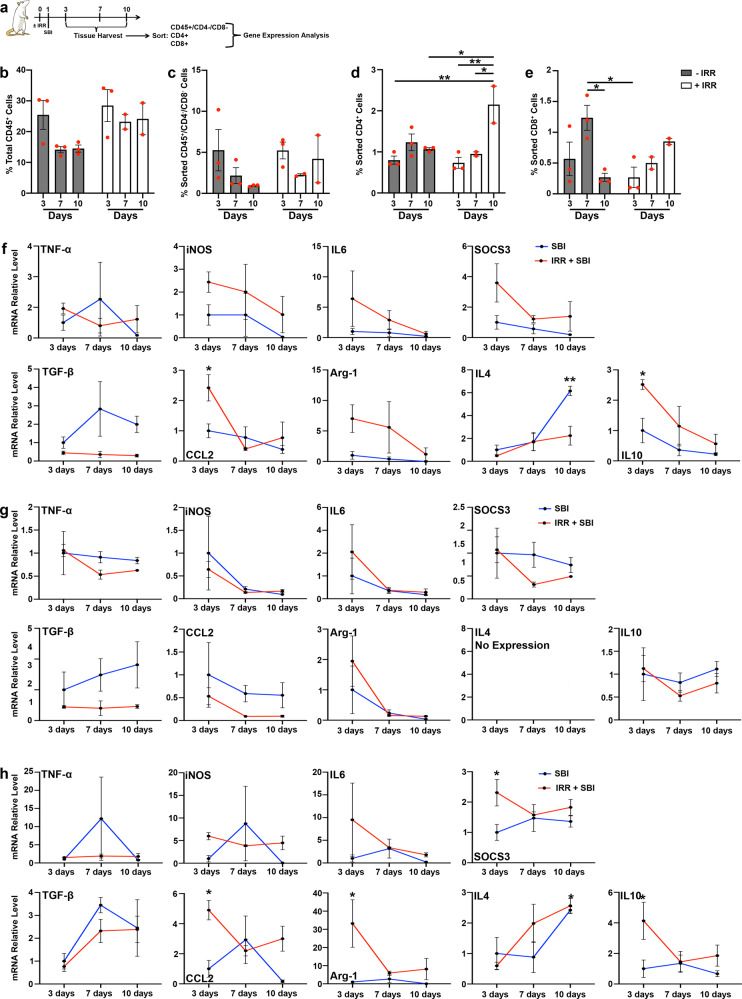
Immune cells gene expression during pelvic floor muscle regeneration in non-irradiated and irradiated animals. **a** Schematic representation of experimental design in panels B-H. **b** Graphical representation of the percentage of CD45^+^ total cells from all the sorted samples. **c** Graphical representation of the percentage of CD45^+^/CD4^−^/CD8^−^ cells sorted for qPCR analysis. **d** Graphical representation of the % of CD4^+^ cells sorted for qPCR analysis. **e** Graphical representation of the percentage of CD8^+^ cells sorted for qPCR analysis. Dots represent single measurements; error bar represent SEM. Two-way ANOVA with Sidak’s post hoc. *n* = 3 animals. **f** qPCR analysis of pro-inflammatory genes (on the top) and of anti-inflammatory genes (on the bottom) for CD45^+^/CD4^−^/CD8^−^ cells. **g** qPCR analysis of pro-inflammatory genes (on the top) and of anti-inflammatory genes (on the bottom) for CD4^−^ cells. **h** qPCR analysis of pro-inflammatory genes (on the top) and of anti-inflammatory genes (on the bottom) for CD8^−^ cells.

Given that irradiation did not impact arterioles, we then asked whether the immune response mounted by irradiated animals after birth injury was comparable to that of nonirradiated group. To address this question, we first isolated three immune populations: CD4^+^ (T-helper cells), CD8^+^ (cytotoxic T-cells), and CD45^+^/CD4^−^/CD8^−^ (comprising all other infiltrating hematopoietic cells except erythrocytes and plasma cells) from non-irradiated and irradiated animals at 3, 7, and 10 days post-SBI (Fig. 4a, Supplementary Fig. 4a, b). We then designed a panel of genes associated with pro- and anti-inflammatory responses of injured skeletal muscles and compared their expression between groups (Fig. 4a, Supplementary Fig. 4a, Supplementary Table 4).

Overall, percentage of immune cells (total CD45^+^ cells) observed during cell sorting (Fig. 4b, e) was similar between non-irradiated and irradiated injured groups 3 days after SBI, indicating that the amount of immune cells recruited into the muscles was not altered by irradiation. However, the decrease in immune cell number detected in non-irradiated injured PCa between days 3 and 10 post-SBI was not observed in the irradiated injured muscles. In contrast to the non-irradiated samples, a greater number of CD4^+^ cells was present 10 days after SBI compared to the earlier time points in the irradiated injured group (Fig. 4d). Similarly, elevated CD8^+^ cell infiltrate persisted at 10 days post-SBI in irradiated injured PCa, while significant decrease in CD8^+^ cells was observed in nonirradiated muscles at this time point (Fig. 4e). Analogous patterns were observed with respect to ICa and C (Supplementary Fig. 4c–f). Taken together, these results suggest that while the number of immune cells acutely recruited by PFMs following birth injury is similar in irradiated and non-irradiated conditions, the irradiated muscles retain elevated immune infiltrate for a longer time after injury than non-irradiated PFMs.

In addition to the quantitative difference in the immune infiltrate described above, CD8^+^ and CD45^+^/CD4^−^/CD8^−^ cells isolated from PCa demonstrated a higher expression of both pro-inflammatory and anti-inflammatory cytokines genes in irradiated samples compared to non-irradiated muscles (Fig. 4f–h). We observed significantly greater CCL2 and IL10 expression in both CD8^+^ and CD45^+^/CD4^−^/CD8^−^ cells, and SOCS3 and Arg1 expression in CD8^+^ cells isolated from irradiated relative to non-irradiated PCa 3 days after SBI (Fig. 4f–h). The only exceptions were in the expression of the anti-inflammatory markers, TGFβ and IL4, which was higher in non-irradiated compared to irradiated injured animals. CD45^+^/CD4^−^/CD8^−^ cells isolated from ICa and C similarly demonstrated increased expression of both pro-inflammatory and anti-inflammatory markers in irradiated compared to non-irradiated muscles. TGFβ expression did not differ between irradiated and non-irradiated ICa and C, while IL4 expression was higher in non-irradiated muscles, as was observed in PCa (Supplementary Fig. 4g–l). Consistent with PCa, CD8^+^ cells from C had increased expression of both pro- and ani-inflammatory genes, with iNOS and IL6 significantly upregulated in cells isolated from irradiated relative to non-irradiated C 7 days after SBI (Supplementary Fig. 4l). Also consistent with PCa, TGFβ expression in CD8^+^ cells did not differ between irradiated and non-irradiated C (Supplementary Fig. 4l). ICa CD8^+^ cells did not show any significant difference between non-irradiated and irradiated groups (Supplementary Fig. 4i). Gene expression of CD4^+^ cells isolated from non-irradiated and irradiated PCa and C did not show any difference at any time point analyzed. In contrast, CD4^+^ cells isolated from ICa had significantly higher iNOS expression in irradiated and significantly higher IL10 expression in non-irradiated muscles 10 days after SBI (Fig. 4g, Supplementary Fig. 4h, and Supplementary Fig. 4k). Overall, our analyses revealed that the immune response to birth injury was enhanced and prolonged, at least up to 10 days, in animals subjected to irradiation before SBI (Fig. 1h). In addition, PCa, ICa, and C differed with respect to gene expression within specific cell type, indicating that the variable extent of injury observed in these individual components of the PFM complex impacts the immune response^22^.

Tibialis anterior (TA), a hind limb muscle, was harvested from both non-irradiated and irradiated animals and the total CD45^+^ population was isolated and processed for gene expression analysis (Supplementary Fig. 4c–m). TA served as nonpelvic control for assessing the systemic effect of irradiation in uninjured condition. There were no significant differences in gene expression of various cytokines in irradiated vs non-irradiated TA. However, TGFβ and IL4 gene expression was significantly upregulated in cells isolated from non-irradiated relative to irradiated TA at the acute time points after SBI, similar to PFMs (Supplementary Fig. 4m).

### Early muscle regeneration is impaired in irradiated regenerating PFMs

**Fig. 5:**
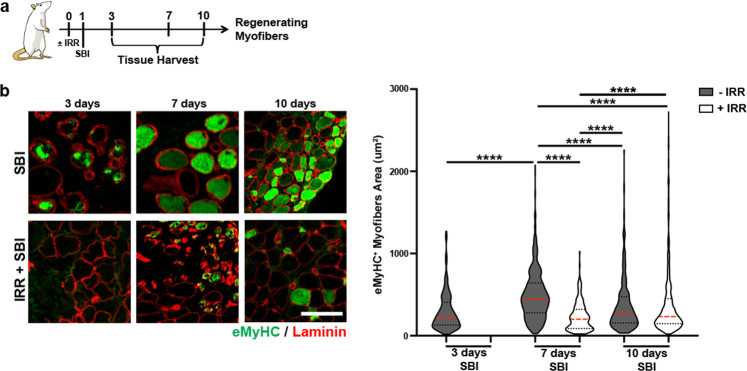
Early pubocaudalis (PCa) muscle regeneration in non-irradiated and irradiated animals. **a** Schematic representation of the experimental design for B. **b** On the left, representative immunofluorescent images of eMyHC/laminin staining (scale bar: 100μm). On the right, violin plots representation of eMyHC^+^ fiber area. ****p value < 0.0001; Kruskal-Wallist with Dunn’s multiple comparison test. *n* = 3 animals.

Given the reduction in MuSC proliferative ability, lack of FAPs expansion, and clear phenotypic differences observed in irradiated vs non-irradiated PFMs 4 weeks after birth injury, we went on to further explore the events during acute and subacute recovery period. Previously, we have demonstrated the presence of embryonic myosin heavy chain (eMyHC) positive fibers in PCa as early as 3 days post-SBI in nonirradiated animals^29^. Using the same anti-eMyHC antibody, we assessed myofiber regeneration at 3, 7, and 10 days postinjury in the irradiated group (Fig. 5a, Supplementary Fig. 5a). In contrast to the observations in non-irradiated animals, eMyHC^+^ fibers were absent in irradiated injured animals at 3 days post-SBI (Fig. 5b). At 7 days, the size of eMyHC^+^ fibers was significantly smaller in irradiated compared to non-irradiated rats (Fig. 5b). The difference in eMyHC^+^ fiber size between irradiated and non-irradiated animals was no longer observed at 10 days postinjury (Fig. 4b). The above findings signify that the regenerative process is delayed in irradiated animals, whereas non-irradiated animals with intact MuSC and FAP function and temporally coordinated immune response start the regenerative process as early as 3 days after injury. Similar trends were observed in the ICa and C muscles (Supplementary Fig. 5b, c). Taken together, these results indicate that deficient MuSC proliferation and lack of FAP expansion together with persistent immune response impair female PFM regeneration following birth injury.

## Discussion

Our novel study examines the functional role of the major players (MuSCs, FAPs, and immune cells) in female PFM regeneration after birth injury. Employing a validated simulated birth injury rat model and irradiation as a tool to perturb skeletal muscle regeneration, we show that PFM fiber size is substantially reduced in irradiated compared to non-irradiated animals 4 weeks after birth injury. Persistent immune response, evidenced from significant increase in interstitial leukocytes, was also observed in irradiated injured PFMs. We demonstrate that in injured non-irradiated animals, FAPs and MuSCs increase in number at 3 and 7 days following SBI, leading to the timely activation of the regenerative process. In contrast, MuSCs and FAPs in irradiated PFMs do not expand during the first stages of muscle regeneration following birth injury. Consequently, regenerating muscle fibers do not appear in irradiated PFMs until day 7 post-SBI, as opposed to day 3 in the non-irradiated injured group. NMJ density and arteriolar size and density were not altered up to 28 days after irradiation. Taken together, these results show that the MuSCs and FAPs expansion as well as the temporal regulation of the immune response are necessary to ensure constructive PFM recovery after birth injury and that the disruption of these processes leads to substantial PFM atrophy.

Previously, we have identified differential response of the individual rat PFMs to mechanical birth injury, owing to variable strains imposed on the individual components of the PFM complex during SBI^22^. Specifically, we have shown that coccygeus (C) and the pubocaudalis (PCa) portion of the rat levator ani muscle are significantly more affected by SBI, as indicated by dramatic myofiber stretch, and sarcomere hyperelongation and misalignment, while the iliocaudalis (ICa) portion of the rat levator ani muscle is less impacted^22^. In the current study, we observed a corresponding variable effect of birth injury on MuSCs and FAPs in the individual components of the rat PFM complex. Specifically, in response to SBI, MuSC number and proliferation increased in C and PCa, but not in ICa. Likewise, FAP number increased acutely after injury only in C and PCa. Notably, we observed large injury-regeneration areas, indicated by the presence of eMyHC^+^ fibers, in these two muscles, whereas the extent of injury was smaller and more variable in ICa. Our discoveries in the rat PFMs are consistent with the findings of computer modeling of human parturition and clinical radiological studies, which show that the iliococcygeus (analogous to ICa) portion of the levator ani muscle experiences less strain than pubococcygeus (analogous to PCa) during fetal delivery^23,24,28^. Unfortunately, coccygeus has been omitted from the majority of the human studies this far. Here we show that parturition-related strains of variable magnitudes imposed on each PFM during SBI correspond to disparate cellular responses to birth injury. Indeed, ICa, which experiences the smallest parturition-associated strains, demonstrates substantially reduced cellular responses to SBI compared to PCa and C. Our findings are also consistent with the investigations conducted in hind limb muscles, where MuSC and FAP increase in number after injury, while cells in uninjured muscles do not change quantitatively over time^7,31–33^.

The existence of multiple transgenic mouse models makes the study of the relative contribution of MuSCs, FAPs, and immune cells to muscle regeneration relatively easy in the murine model^3,34–37^. However, the mouse model has been shown to be less well-suited than the rat model for the studies of the human PFMs^21,38^. Thus, given the lack of rat genetic models, we used irradiation as a tool to determine the role of MuSCs, FAPs, and immune cells in female PFM regeneration. It has been previously shown that the MuSC compartment in hind limb muscles is affected by irradiation. Indeed, radiation induces accumulation of DNA damage in MuSCs, in turn, inhibiting cellular capacity to proliferate and differentiate into mature myofibers after injury^26,39,40^. Moreover, MuSC self-renewal ability is also reduced by irradiation^26^. Consistent with previous observations in appendicular muscles, we demonstrate that the impairment of PFM stem cells’ ability to enter cell cycle and proliferate correlates with a delay in the formation of regenerating myofibers and a reduction in their cross-sectional area during early regeneration.

The explicit effect of irradiation on FAPs has not been previously described^41,42^. Nevertheless, it has been hypothesized that because FAPs become activated in response to muscle injury, their function could be compromised by irradiation^7,41^. Here, we provide direct evidence that the function of FAPs is negatively affected by irradiation. Given the major role that FAPs play in regulating the activation and proliferation of MuSCs, the loss of function in these cells in our model system may contribute to the observed defect in PFMs’ repair. FAPs are also a major source of intramuscular collagen and fat. Irradiation-induced impairment in FAPs’ ability to activate in response to birth injury might partially account for the absence of a higher fibrotic and fatty degeneration in irradiated relative to non-irradiated injured PFMs 4 weeks after SBI^43^. However, we cannot exclude the possibility that pathological deposition of collagen originating from other sources might continue in irradiated PFMs, leading to worsening fibrosis at a later time point, as has been previously shown in other muscles in both human and animal studies^44,45^. We also cannot rule out that persistent inflammation, observed in irradiated PFMs following birth injury, does not change FAPs’ fate and drive their differentiation into adipocytes at later time, similarly to what happens in other pathological conditions^46^.

Studies aiming at determining the effect of irradiation on inflammatory response in regenerating muscles are also limited^41,47^. When a healthy muscle was injured 24 h after local irradiation, no difference in the amount of infiltrating cells was observed during the early regenerative process^47^. However, in a chronic inflammatory muscle environment, such as muscular dystrophy, irradiation has been shown to increase the innate immune response^41^. We found that the initial immune infiltrate in PFMs 3 days after birth injury does not differ between non-irradiated and irradiated animals, but that the immune response is significantly increased and persists up to 10 days in irradiated injured animals. Indeed, with the exception of TGFβ and IL4, the expression of pro- and anti-inflammatory genes was persistently increased in cells isolated form irradiated animals throughout PFMs’ recovery. MuSC and FAPs’ loss of function and the associated impairment of PFM regeneration expose immune cells to an aberrant environment. Consequently, immune cells lose their ability to coordinate temporally regulated muscle regeneration and persist in PFMs further contributing to their impaired recovery. Indeed, 4 weeks after birth injury we still observe significantly higher number of infiltrating immune cells in PFMs isolated from irradiated animals compared to all other conditions.

To evaluate potential effects of irradiation on various muscle components, we assessed NMJs and arterioles of the PFMs. Investigations on the effects of irradiation on skeletal muscle innervation are limited and have been mainly performed in the context of nerve crush injury, thus little is known about the effect of irradiation on NMJs. Previous studies conducted in soleus muscle showed no effect of irradiation on muscle innervation, evaluated through functional measures, 3 to 4 months after treatment with a 16 Gy dose^48^. In the current study, we did not observe an effect of irradiation on NMJs density or any associated changes in the myofiber CSA up to 28 days after irradiation. Likewise, irradiation did not affect either the size or the density of intramuscular arterioles up to one month after irradiation. Similarly to our finding, a single high dose irradiation of dystrophic animals has been shown not to affect endothelial cells up to 28 days after treatment^40^. Despite our extensive analysis and the fact that post-mitotic myofibers have been shown to be radiation-resistant^49^, we cannot exclude that irradiation could affect myoblasts, resident macrophages, or pericytes, as these were not directly evaluated in the current study.

Interestingly, despite employing a high-dose pelvic gamma radiation that negatively affected proliferation of pelvic muscles’ MuSCs and FAPs, we still observed a certain level of muscle regeneration. Previous studies have demonstrated that a subpopulation of MuSCs is capable of forming fully differentiated myofibers upon transplantation despite 18 Gy irradiation^40^. It is also known that this MuSC subpopulation is activated in response to injury^50^. Moreover, it has been recently reported that radiation-resistant MuSCs have enhanced Pax3 expression and can expand in vivo after injury despite irradiation, contributing to muscle regeneration^51^. Pax3 expression is necessary for protection of cells from external stresses, preventing premature cellular activation and cell death through activation of G-alert state, characterized by accelerated cell cycle entry^52^. Exploring whether incomplete loss of the PFMs’ regenerative capacity in the irradiated injured group is due to the presence of radiation-resistant MuSCs is a fruitful avenue for the future studies.

Inadequate PFM repair following birth injury, observed in the current study, led to the pathological alterations (myofiber atrophy and fibrotic degeneration) in rat PFMs that mimic the PFM derangements observed in women with pelvic floor disorders. Evidence that such pathological alterations are responsible for PFM dysfunction in humans comes from our recent findings that the pubovisceralis (analogous to rat pubocaudalis) muscle of parous women with symptomatic pelvic organ prolapse exhibits widespread dramatic myofiber degeneration and fibrosis, not evident in PFMs from age-matched nulliparous cadaveric donors without pelvic floor disorders^29^. The PFM phenotype observed 4 weeks after birth injury in the irradiated animals, encompassed similar pathological transformations to those induced by SBI alone, but with greater severity of these untoward alterations.

In conclusion, the current study represents unprecedented work focused on perturbing MuSCs, FAPs, and immune response during female PFM regeneration after birth injury, the leading risk factor for the development of morbid and prevalent pelvic floor disorders. We demonstrate that PFM constructive regeneration following birth injury is dependent on MuSC and FAP ability to become activated and proliferate, as well as on the temporally-regulated immune response. Future studies will aim to determine the specific role of MuSCs during regeneration through cell transplantation experiments performed in irradiated animals before or after birth injury. In addition, given that persistent immune response could negatively affect PFM regeneration, immunomodulatory drugs can be used to reduce the immune response starting 7 days after injury to mimic decreased inflammation observed in non-irradiated regenerating muscles. Further perturbing this model would allow us to definitively determine whether MuSCs, FAPs or immune cells could be used as actionable therapeutic targets to enhance the endogenous regenerative potential of the PFMs. Such strategies, which are lacking to date, would greatly improve outcomes in women at high risk for maternal pelvic floor injury (operative vaginal delivery, macrosomia, prolonged second stage of labor) or in women with potentially diminished ability for adequate PFM recovery following birth injury (older maternal age, extensive muscle injury, previous substantial muscle injuries, compromised immune response).

## Methods

### Animals

Female 3-month old Sprague-Dawley rats (Envigo, Indianapolis, IN) were randomly divided in 4 study groups: (1) unperturbed controls; (2) animals subjected to simulated birth injury (SBI) only; (3) animals undergoing irradiation only; and (4) animals subjected to irradiation and SBI. The rats were allowed to recover for either 3, 7, 10, or 28 days post SBI, at which point they were euthanized by CO_2_ inhalation followed by bilateral thoracotomy (*N* = 3/group/time point). Bilateral coccygeus (C), iliocaudalis (ICa), pubocaudalis (PCa), and nonpelvic control muscle - tibialis anterior (TA)- were harvested immediately following euthanasia. All procedures were approved by the University of California San Diego Institutional Animal Care and Use Committee and all the experiments were performed in accordance with Institutional Animal Care and Use Committee guidelines.

### Simulated birth injury (SBI)

SBI was performed using the established vaginal distention protocol, as described in Alperin et al. 2010^27^. Briefly, rats were anesthetized with 2.5% isoflurane with oxygen for the duration of the procedure. A 12-French transurethral balloon catheter (Bard Medical, Covington, GA) with the tip cut off was inserted into the vagina with 130 grams weight attached to the end of the catheter. The balloon was inflated to 5 ml and left in place for 2 h, after which it was pulled through the introitus to simulate the circumferential and downward distention associated with fetal crowning and parturition.

### BaCl_2_ injury

BaCl_2_ (10 µL) was injected into the enthesial portion of the pubocaudalis muscle via transobturator approach employing a 30 G needle on a Hamilton syringe. This approach was validated as a reliable method for minimally invasive delivery of injectable substances to PCa^53^.

### Irradiation

Animals were irradiated at UCSD animal facility with the Small Animal IGRT Platform (SmART+) from Precision X-Ray Irradiation using SmART Advanced Treatment Planning software. Pelvic floor and hind limb muscles were irradiated with a single dose of 20 Gy (225 kV, 20 mA with a treatment filter).

### Immune cells isolation

Muscles (PCa, ICa, C, and TA) were digested using the same protocol as in our previous studies^54^. Anti CD45 (BD Biosciences, 565465, [0.3 ug/10^6^ cells]), CD4 (Invitrogen, 11-0040-81, [0.2 ug/10^6^ cells]), and CD8 (Biolegend, 200608, [0.2 ug/10^6^ cells]) antibodies were used to identify general immune cells population, T-helper cells, cytotoxic T-cells, respectively. All antibodies were first tested and titrated to determine the proper concentration to use for cell isolation (data not shown). Immune cells were isolated with FACSAria II and FACSAria Fusion (BD Biosciences, USA) cell sorters.

### RNA isolation and quantitative PCR

RNA was isolated from immune cells using the RNeasy Micro Kit (QIAGEN, 217084) following the manufacturer’s protocol, and quantified with Qubit RNA assay HS kit (ThermoFisher, Q32855). cDNA was synthesized using the SuperScript™ IV First-Strand Synthesis System (ThermoFisher Scientific, 18091050), and RT-qPCR was performed using Power SYBR^TM^ Green PCR Master Mix (ThermoFisher Scientific, 4367659), on CFX96 Touch™ Real-Time PCR Detection System (Bio-Rad, 1855195) with 0.5 ng cDNA. Primers are listed in Supplementary Table 4. The control gene used was 60 S acidic ribosomal protein P0 (Rplp0) and Ct values for all genes were divided by the Rplp0 Ct value to obtain relative gene expression. Ct values for IL4 were over 35 in CD4^+^ cells from all PFMs, thus this gene was excluded from analysis. Arg1 and IL4 Ct values were over 35 in CD8^+^ cells isolated from C, thus these genes were excluded from analysis.

### Immunostaining

Snap-frozen muscles were sectioned into 10 µm thick slices and fixed for immunostaining in either acetone (Fisher Scientific, A16P-4), 4% PFA (Fisher Scientific, 04042), 2% PFA, or left unfixed depending on the primary antibody used. Slides allocated to the examination of eMyHC, α-SMA, perilipin, CD45, and synaptotagmin2 were incubated in sequence with PBS (phosphate buffered saline) and blocking buffer (20% normal goat serum (Gemini Bio-products, 100–109) + 0.3% Triton X-100 (Sigma-Aldrich, X100-500ML) in PBS or 1% BSA (Cytiva, SH30574.01) + 0.05% Triton X-100 in PBS) before overnight incubation with primary antibody. For Pax7 and Ki67 staining, slides were washed in PBS and then placed in antigen unmasking solution (Vector Laboratories, H-3301-250) for antigen retrieval. Sections were washed with PBS and incubated in a blocking buffer before adding the primary antibody. Slides stained with anti-PDGFR-α antibody were first washed with PBS, then incubated with a 0.5% Triton X-100 solution and finally with casein blocking buffer (0.25% casein (Sigma, C3400-500G) in PBS) before overnight incubation with primary antibody. Slides stained for collagen I were first washed and then incubated in blocking buffer (10% goat serum, 0.3% Triton x-100, 1% BSA (Gemini Bio-Products, 700–100 P) in PBS) before overnight incubation with primary antibody. Slides stained for CD68 were blocked with 1%BSA in PBS before incubation with primary antibody overnight. All slides were washed in PBS (+0.1% Triton X-100 for CD68 staining) and then incubated with secondary antibody in a blocking buffer for 1 h. To identify cell nuclei, slides were incubated with DAPI (ThermoFisher Scientific, 62248, 1:1000) in PBS for 10 min.

Primary antibodies used included: rabbit anti-laminin (Sigma, L9393, 1:200), mouse anti- eMyHC (Developmental Studies Hybridoma Bank (DSHB), F1.652, 1:200), mouse anti-Pax7 (Developmental Studies Hybridoma Bank (DSHB), Pax7-c, DSHB, 1:100), and rabbit anti-Ki67 (Abcam, ab15580, 1:100), goat anti-PDGFR-α (R&D Systems, AF1062, 1:100), α-SMA (Cell Signaling, 19245 S, 1:100), collagen I (Invitrogen, PA5-95137, 1:200), CD45 (1:100), CD68 (Biorad, MCA341GA, 1:100), Perilipin (Fisher, PA1-1052, 1:200), synaptotagmin (Developmental Studies Hybridoma Bank (DSHB), znp-1, 1:500), bungarotoxin (Invitrogen, B13422, 1:250). Secondary antibodies included: Alexa Fluor 488 goat anti-mouse IgG (Invitrogen, A21121, 1:200 for eMyHC and 1:250 for Pax7), Alexa Fluor 546 goat anti-mouse IgG (Invitrogen, A11030, 1:250), Alexa Fluor 546 goat anti-rabbit IgG (Invitrogen, A11035, 1:500 for laminin and 1:250 for Ki67), Alexa Fluor 546 donkey anti-goat IgG (Invitrogen, A-11056, 1:250), Alexa Fluor 488 donkey anti-rabbit (Invitrogen, A-21206, 1:250).

### Hematoxylin and eosin staining

Snap frozen tissue sections were first rinsed in water, then immerse in Harris’s Hematoxylin for 3 min, followed by water rinses and 30 dips in lithium carbonate solution. Slides where then dip in 1% eosin solution in acetic acid, and de-hydrated in ethanol. Slides where finally dipped in Citri-Soly until they became clear and finally protected with a coverslip.

### Imaging

Immunofluorescence imaging was carried out using the LEICA fluorescent microscope (LEICA AF6000 Modular System) and Keyence BZX800. Slides stained for fibrosis were imaged with a Leica Aperio ScanScope® CS2.

### Quantification

#### Embryonic myosin heavy chain (eMyHC)

for each PFM (PCa, ICa, or C) harvested from each injured animal, 1 tissue section containing the largest observable area of injury from either unilateral or bilateral muscles were chosen for analysis. Full images of each section were captured at 10X and the cross-sectional area of each eMyHC^+^ myofiber was measured. Myofibers cross sectional area was assessed using ImageJ 1.51 s and ImageJ64. Images were not modified before quantification.

#### Pax7/ki67

for each rat muscle section, 3 images were taken at 20X of each tissue section, from both the right and left sides. The number of Pax7^+^ cells, and Pax7^+^/ki67^+^ cells were counted in each image. The number of Pax7^+^ cells was calculated per unit area, while Pax7^+^/ki67^+^ double positive cells were expresses as percentage. Unmodified images were analyzed using ImageJ 1.51 s and Adobe Photoshop CS4.

#### Pdgfr-α

for each uninjured muscle section, 3 images were taken at 20X of each tissue section, from both the right and left sides. The number of PDGFR- α ^+^ cells, and cells were counted in each image. For injured muscle, only the sections containing an injury were imaged at 20X and used for quantification. The number of PDGFR-α^+^ cells was calculated per unit area. Unmodified images were analyzed using ImageJ 1.51 s and Adobe Photoshop CS4.

#### Collagen

one slide per muscle sample, containing 10–14 tissue sections, was analyzed employing ImageJ 1.51 s. Specifically, all sections were outlined with the freehand selections tool (edges of the muscle section, nerves, and large blood vessels were excluded from the analysis), converted into binary images, and quantified as percentage area occupied by collagen staining.

#### Perilipin

for each muscle section (8–10 sections per slide), 3 images were taken at 20X. Analysis was performed with ImageJ 1.53k. Perilipin^+^ areas were identified using the MaxEntropy thresholding method, areas of positive staining were highlighted with wand tool and divided by the total area of the muscle cross-section.

#### CD45, CD68

for each slide, 4 randomly selected whole tissue sections (2 from the right and 2 from the left muscle) were analyzed with ImageJ 1.53k. All the sections were outlined with a freehand tool to determine the area of the muscle, and all the interstitial cells positive for the respective staining within the defined area were quantified and expressed as number of cells per area.

#### Neuromuscular Junctions

neuromuscular junctions were defined as colocalization of pre- (synaptotagmin) and post-synaptic (α-bungarotoxin) markers. Cross-sectionally cut tissues were used for the quantification, which was performed using Image J software. NMJ density is calculated as number of NMJ per mm^2^.

### Statistical Analysis

Data were analyzed using GraphPad Prism v8.0, San Diego, CA. Distribution was assessed using Kolmogorov-Smirnov test for normality. Data that followed a parametric distribution, such as collagen content, MuSC number and percentage of proliferating cells, FAP number, and blood vessels size and density were compared using one-analysis of variance (ANOVA) followed by post hoc pairwise comparisons using either Tukey’s or Sidak’s tests, when indicated. Non-parametrically distributed variables, such as fiber area, were analyzed by Kruskal-Wallis test followed by Dunn’s pairwise comparisons (for eMyHC^+^ fibers) or with Mann-Whitney test (for 28 days after injury time point). Gene expression data were analyzed using a two-way ANOVA with Dunnett’s post-hoc test with a significance set to *p* < 0.05.

### Reporting summary

Further information on research design is available in the Nature Research Reporting Summary linked to this article.

## Supplementary information

Supplementary Information

Reporting Summary

## Data Availability

Data are available upon request
