# Supplementary material for: Muscle stem cells and fibro-adipogenic progenitors in female pelvic floor muscle regeneration following birth injury
Source: NPJ Regen Med. 2022 Dec 16;7:72. doi: 10.1038/s41536-022-00264-1 (PMC9758192; doi:10.1038/s41536-022-00264-1)
Supplement: Supplementary file 1 — Supplementary Information [file 41536_2022_264_MOESM1_ESM.pdf]

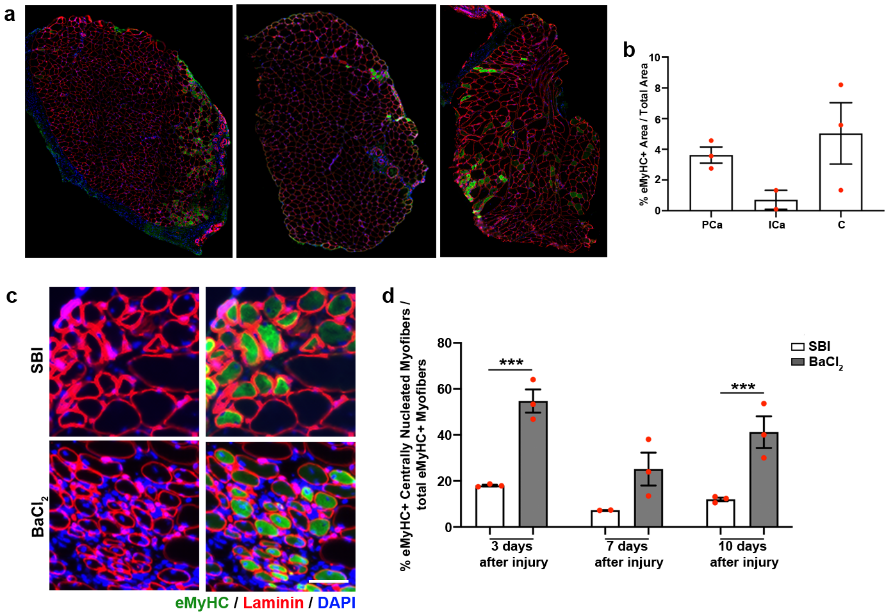
a
b
% eMyHC+ Area / Total Area
10
8
6
4
2
0
PCa
ICa
C
c
SBI
BaCl2
eMyHC / Laminin / DAPI
d
% eMyHC+ Centrally Nucleated Myofibers / total eMyHC+ Myofibers
80
60
40
20
0
***
***
SBI
BaCl2
3 days after injury
7 days after injury
10 days after injury

Supplementary Figure 1 – *Iliocaudalis (ICa) and coccygeus (C) response to injury in non-irradiated and irradiated animals*

(**a**) Representative immunofluorescent images of eMyHC/laminin staining in PCa, ICa, and C 7 days after injury. (**b**) Quantification of percentage of eMyHC$^+$ areas over the total muscle section area. Colored dots represent single measurements; error bar represent SEM. One-way ANOVA with Tukey's post-hoc. n=2-3. (**c**) Representative immunofluorescent images of eMyHC/laminin/DAPI staining in PCa muscle 7 days after SBI injury (top) and $BaCl_2$ injury (bottom). (**d**) Quantification of percentage of eMyHC$^+$ centrally nucleated myofibers in PCa muscles injured with SBI or with $BaCl_2$. **: p-value < 0.01; ***: p-value< 0.001; Two-way ANOVA with Sidak's multiple comparison test. n=2-3.

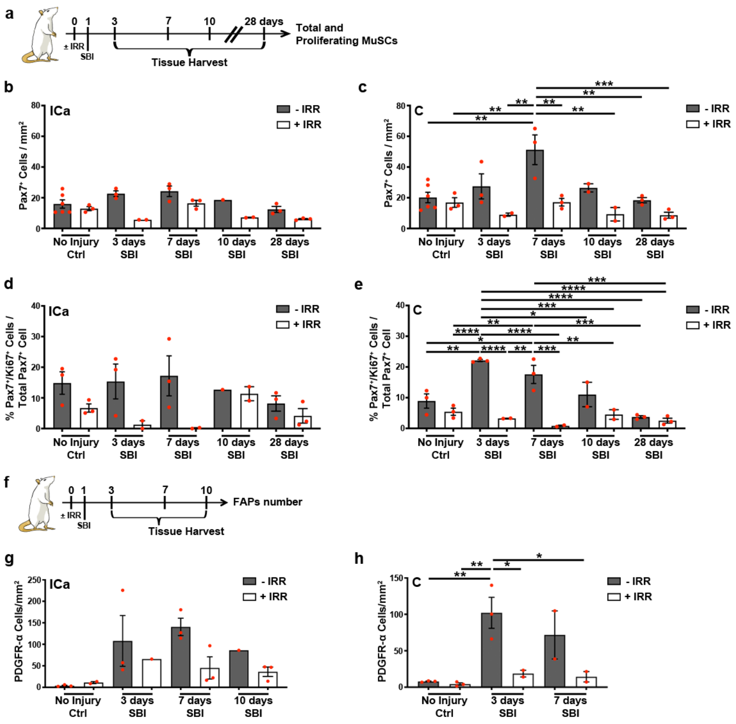

a
0 1 3 7 10 28 days
± IRR
SBI
Tissue Harvest
Total and
Proliferating MuSCs
b
ICa
- IRR
+ IRR
Pax7⁺ Cells / mm²
No Injury Ctrl
3 days SBI
7 days SBI
10 days SBI
28 days SBI
c
C
- IRR
+ IRR
Pax7⁺ Cells / mm²
d
ICa
% Pax7⁺/Ki67⁺ Cells / Total Pax7⁺ Cell
e
C
% Pax7⁺/Ki67⁺ Cells / Total Pax7⁺ Cell
f
0 1 3 7 10
± IRR
SBI
Tissue Harvest
FAPs number
g
ICa
PDGFR-α Cells/mm²
h
C
PDGFR-α Cells/mm²

Supplementary Figure 2 – *Muscle stem cells (MuSCs) and fibro-adipogenic progenitors (FAPs) behavior in regenerating Iliocaudalis (ICa) and coccygeus (C) muscles in non-irradiated and irradiated animals*

(**a**) Schematic representation of the experimental design for b to e. (**b-e** and **g-h**) Red dots represent single measurements; error bar represents SEM. (**b**) Bar graph representing the quantification of MuSCs number per mm^2 in ICa muscle. One-way ANOVA with Tukey's post-hoc. n=3 (**c**) Bar graph representing the quantification of MuSCs number per mm^2 in C muscle. *: p-value < 0.05; **: p-value < 0.01; ***: p-value < 0.001; One-way ANOVA with Tukey's post-hoc. n=3 (**d**) Bar graph representing the quantification of Pax7/Ki67 double positive cells in ICa muscle. One-way ANOVA with Tukey's post-hoc. n=3 (**e**) Bar graph representing the quantification of Pax7/Ki67 double positive cells in C muscle. *: p-value < 0.05; **: p-value < 0.01; ***: p-value < 0.001; ****: p-value< 0.0001; One-way ANOVA with Tukey's post-hoc. n=3. If the number of red dots within one sample is <3, it means that one of the analyzed samples did not show injured area and thus could not be quantified. (**f**) Schematic representation of the experimental design for g and h. (**g**) Bar graph representing the quantification of FAPs number per mm^2 in ICa muscle. (**h**) Bar graph representing the quantification of FAPs number per mm^2 in C muscle. *: p-value < 0.05; **: p-value < 0.01; One-way ANOVA with Tukey's post-hoc. n=3.

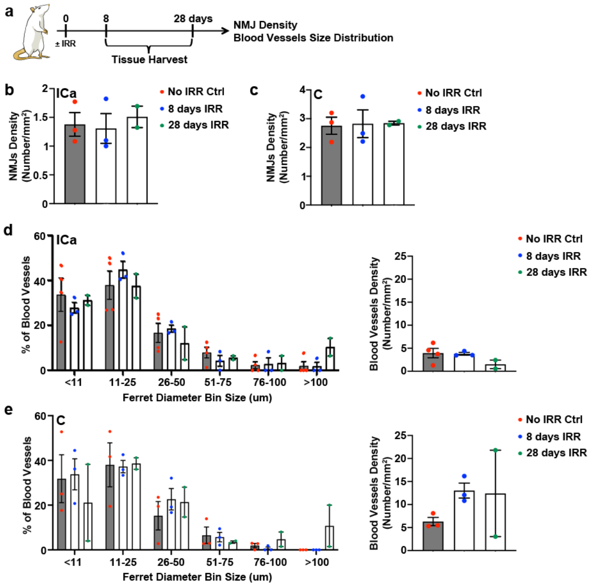
a
0
± IRR
8
28 days
Tissue Harvest
NMJ Density
Blood Vessels Size Distribution
b
ICa
NMJs Density (Number/mm²)
No IRR Ctrl
8 days IRR
28 days IRR
c
C
NMJs Density (Number/mm²)
No IRR Ctrl
8 days IRR
28 days IRR
d
ICa
% of Blood Vessels
<11
11-25
26-50
51-75
76-100
>100
Ferret Diameter Bin Size (um)
Blood Vessels Density (Number/mm²)
No IRR Ctrl
8 days IRR
28 days IRR
e
C
% of Blood Vessels
<11
11-25
26-50
51-75
76-100
>100
Ferret Diameter Bin Size (um)
Blood Vessels Density (Number/mm²)
No IRR Ctrl
8 days IRR
28 days IRR

Supplementary Figure 3 – *Neuromuscular junctions and blood vessels in ICa and C muscles from irradiated uninjured animals*

(**a**) Schematic representation of the experimental design for b and e. (**b-e**) Colored dots represent single measurements; error bars represent SEM. (**b**) Bar graph representing NMJ density in ICa muscle. (**c**) Bar graph representing NMJ density in C muscle. (**d**) Bar graph representing the distribution of blood vessel ferret diameters in ICa muscle. and bar graph representing blood vessel density. One-way ANOVA with Tukey's post-hoc. (**e**) Bar graph representing the distribution of blood vessel ferret diameters in C muscle and bar graph representing blood vessel density. One-way ANOVA with Tukey's post-hoc.

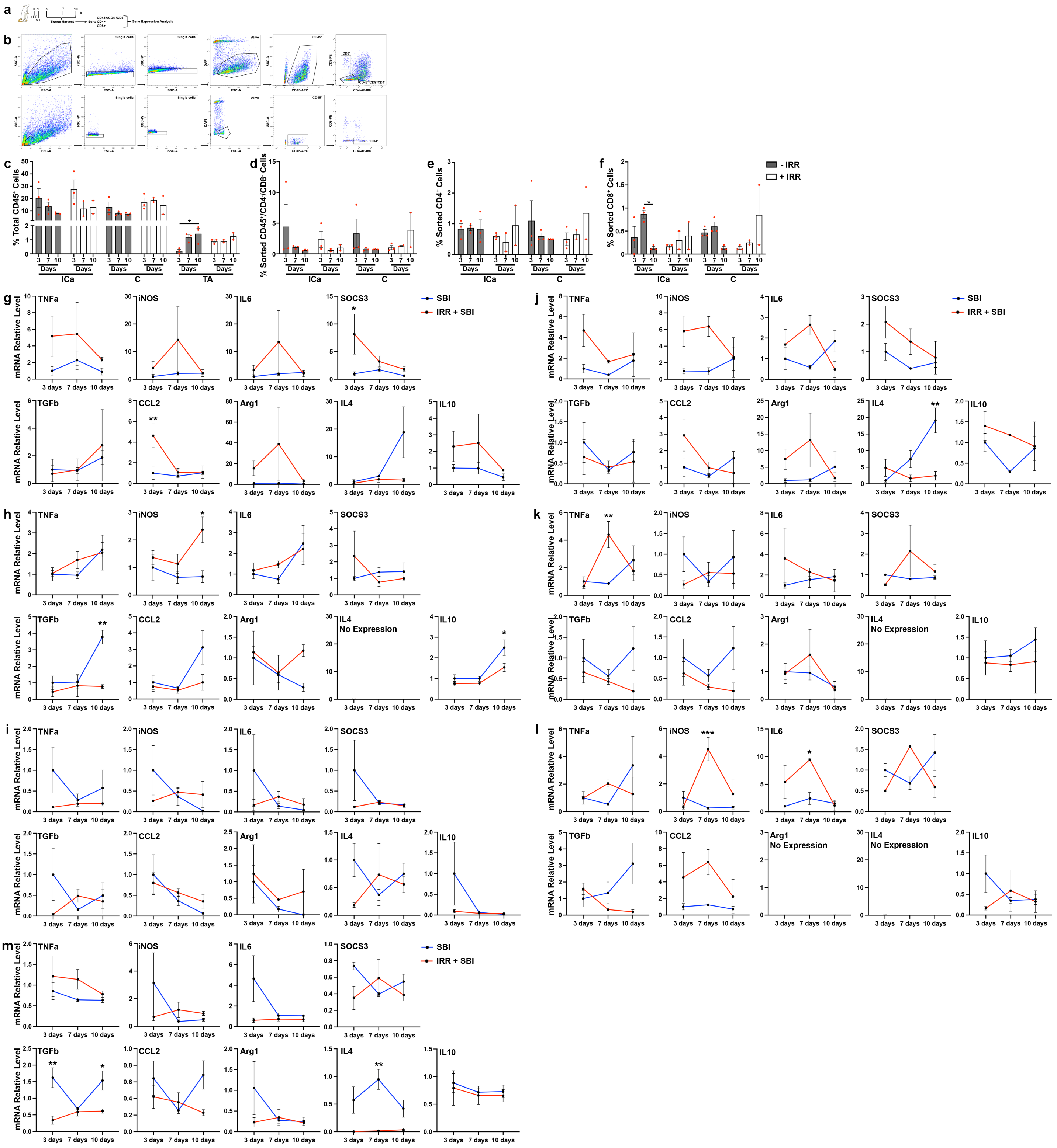
a
Tissue Harvest
Sort: CD4+ CD8+
CD45+/CD4-/CD8-
Gene Expression Analysis
b
FSC-A
SSC-A
FSC-W
SSC-W
DAPI
CD45-APC
CD8-PE
CD4-AF488
Single cells
Alive
CD45+
CD8+
CD45+/CD8-/CD4-
CD4+
c
% Total CD45+ Cells
Days
ICa
C
TA
d
% Sorted CD45+/CD4-/CD8- Cells
e
% Sorted CD4+ Cells
f
% Sorted CD8+ Cells
- IRR
+ IRR
g
h
i
j
k
l
m
mRNA Relative Level
TNFa
iNOS
IL6
SOCS3
TGFb
CCL2
Arg1
IL4
IL10
SBI
IRR + SBI
3 days
7 days
10 days
No Expression

Supplementary Figure 4 – *Immune infiltrate in regenerating Iliocaudalis (ICa), coccygeus (C) and Tibialis Anterior (TA) muscle in non-irradiated and irradiated animals*

(**A**) Schematic representation of the experimental design for b and m. (**B**) FACS plots summarizing the gating system used for sorting immune cells. Top for $CD8^+$ and $CD45^+/CD8^-/CD4^-$ cells; bottom for $CD4^+$ cells. **(C)** Graphical representation of the percentage of $CD45^+$ total cells from all the sorted ICa, C, and TA samples. **(D)** Graphical representation of the percentage of $CD45^+/CD4^-/CD8^-$ cells sorted from ICa, and C for qPCR analysis. **(E)** Graphical representation of the % of $CD4^+$ cells sorted from ICa, and C for qPCR analysis**. (F)** Graphical representation of the percentage of $CD8^+$ cells sorted from ICa, and C for qPCR analysis. Dots represent single measurements; error bar represent SEM. Two-way ANOVA with Sidak's post-hoc. n=3 animals. (**G**) On the top, qPCR analysis of pro-inflammatory genes for $CD45^+/CD8^-/CD4^-$ cells from ICa muscle. On the bottom qPCR analysis of anti-inflammatory genes for $CD45^+/CD8^-/CD4^-$ cells from ICa muscle. Two-way ANOVA with Dunnett's multiple comparison test. n=3 animals. (**H**) On the top, qPCR analysis of pro-inflammatory genes for $CD4^+$ cells from ICa muscle. On the bottom qPCR analysis of anti-inflammatory genes for $CD4^+$ cells from ICa muscle. Two-way ANOVA with Dunnett's multiple comparison test. n=3 animals. (**I**) On the top, qPCR analysis of pro-inflammatory genes for $CD8^+$ cells from ICa muscle. On the bottom qPCR analysis of anti-inflammatory genes for $CD8^+$ cells from ICa muscle. *: p-value < 0.05; **: p-value < 0.01; Two-way ANOVA with Dunnett's multiple comparison test. n=3 animals. (**J**) On the top, qPCR analysis of pro-inflammatory genes for $CD45^+/CD8^-/CD4^-$ cells from C muscle. On the

bottom qPCR analysis of anti-inflammatory genes for CD45$^+$/CD8$^-$/CD4$^-$ cells from C muscle. Two-way ANOVA with Dunnett's multiple comparison test. n=3 animals. (**K**) On the top, qPCR analysis of pro-inflammatory genes for CD4$^+$ cells from C muscle. On the bottom qPCR analysis of anti-inflammatory genes for CD4$^+$ cells from C muscle. Two-way ANOVA with Dunnett's multiple comparison test. n=3 animals. (**L**) On the top, qPCR analysis of pro-inflammatory genes for CD8$^+$ cells from C muscle. On the bottom qPCR analysis of anti-inflammatory genes for CD8$^+$ cells from C muscle. *: p-value < 0.05; **: p-value < 0.01; Two-way ANOVA with Dunnett's multiple comparison test. n=3 animals. (**M**) On the top, qPCR analysis of pro-inflammatory genes for all CD45$^+$ cells from TA muscle. On the bottom qPCR analysis of anti-inflammatory genes for all CD45$^+$ cells from TA muscle. Two-way ANOVA with Dunnett's multiple comparison test. n=3 animals.

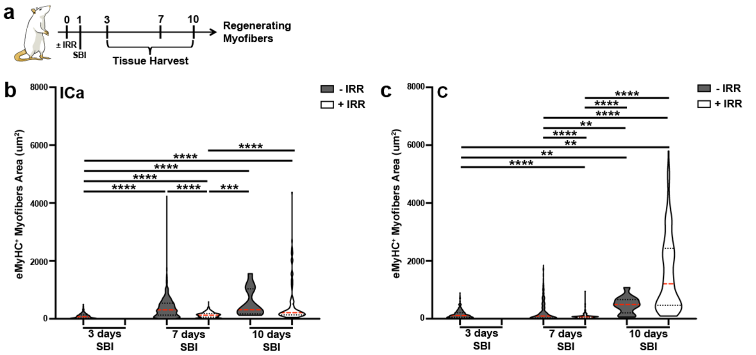

Figure S5 – Early Iliocaudalis (ICa) and coccygeus (C) muscles regeneration in non-irradiated and irradiated animals
(A) Schematic representation of the experimental design for B and C. (B) Violin plots representation of eMyHC+ fiber area for ICa muscle. ***: p-value < 0.001; ****: p-value< 0.0001; Kruskal-Wallist with Dunn's multiple comparison test. (C) Violin plots representation of eMyHC+ fiber area for C muscle. **: p-value < 0.01; ****: p-value< 0.0001; Kruskal-Wallist with Dunn's multiple comparison test.

Supplementary Figure 5 – *Early Iliocaudalis (ICa) and coccygeus (C) muscles regeneration in non-irradiated and irradiated animals*

(**a**) Schematic representation of the experimental design for b and c. (**b**) Violin plots representation of eMyHC$^+$ fiber area for ICa muscle. ***: p-value < 0.001; ****: p-value< 0.0001; Kruskal-Wallis with Dunn's multiple comparison test. (**c**) Violin plots representation of eMyHC$^+$ fiber area for C muscle. **: p-value < 0.01; ****: p-value< 0.0001; Kruskal-Wallist with Dunn's multiple comparison test.

Table S1. Complete statistic for Figure 2b

**Tukey's multiple comparisons test**	**Significance**
No Injury Ctrl -IRR vs 7 days SBI -IRR	****
No Injury Ctrl -IRR vs 10 days SBI -IRR	**
No Injury Ctrl +IRR vs 7 days SBI -IRR	****
No Injury Ctrl +IRR vs 10 days SBI -IRR	**
3 days SBI -IRR vs 7 days SBI	****
3 days SBI -IRR vs 10 days SBI	**
3 days SBI +IRR vs 7 days SBI	****
3 days SBI +IRR vs 10 days SBI	**
7 days SBI -IRR vs. 7 days SBI + IRR	****
7 days SBI -IRR vs 10 days SBI + IRR	****
7 days SBI -IRR vs 28 days SBI	****
7 days SBI -IRR vs 28 days SBI +IRR	****
7 days SBI +IRR vs 10 days SBI	**
10 days SBI -IRR vs 10 days SBI + IRR	**
10 days SBI -IRR vs 28 days SBI	**
10 days SBI -IRR vs 28 days SBI +IRR	**

Table S2. Complete Statistic for figure 2c

**Tukey's multiple comparisons test**	**Significance**
No Injury Ctrl -IRR vs 3 days SBI -IRR	**
No Injury Ctrl -IRR vs 7 days SBI -IRR	*
No Injury Ctrl -IRR vs 10 days SBI -IRR	**
No Injury Ctrl +IRR vs 3 days SBI -IRR	*
No Injury Ctrl +IRR vs 10 days SBI -IRR	*
3 days SBI -IRR vs 7 days SBI +IRR	**
3 days SBI -IRR vs 10 days SBI +IRR	*
3 days SBI -IRR vs 28 days SBI +IRR	**
3 days SBI +IRR vs 10 days SBI -IRR	*
7 days SBI -IRR vs 28 days SBI +IRR	*
7 days SBI +IRR vs 10 days SBI -IRR	*
10 days SBI -IRR vs 28 days SBI +IRR	*

Table S3. Complete statistic for figure 2e

Tukey's multiple comparisons test	Significance
No Injury Ctrl -IRR vs 3 days SBI -IRR	****
No Injury Ctrl -IRR vs 7 days SBI -IRR	****
No Injury Ctrl +IRR vs 3 days SBI -IRR	****
No Injury Ctrl +IRR vs 7 days SBI -IRR	****
3 days SBI vs 7 days SBI +IRR	****
3 days SBI vs 10 days SBI -IRR	**
3 days SBI vs 10 days SBI +IRR	****
3 days SBI+ IRR vs 7 Days SBI -IRR	***
7 days SBI vs 10 days SBI -IRR	**
7 days SBI vs 10 days SBI +IRR	****

Table S4. Primer list

Gene	Forward Primer	Reverse Primer
Pax7	CTCCAAGATTCTGTGCCGGT	ATCGAACTCACTGAGGGCAC
Myf5	GGAATGCAATCCGCTACATT	CAGGGCAGTAGATGCTGTCA
MyoD	TACCCAAGGTGGAGATCCTG	CATCATGCCATCAGAGCAGT
Myogenin	TGGGCGTGTAAGGTGTGTAA	AGGCGCTCAATGTACTGGAT
Myf6	GGGTGGACCCTTACAGCTAC	TCCACGATGGAAGAAAGGCG
MYH3	AGGAGACGCAGAGAAGCCTA	CCACTCGGCTAACACCTTGT
IL6	TAGTCCTTCCTACCCCAATTTCC	TTGGTCCTTAGCCACTCCTTC
TNF-α	ACTGAACTTCGGGGTGATCG	GCTTGGTGGTTTGCTACGAC
TGF-β1	ATTGCTGGGCAAGTGGTTAC	TTATGTCGGATGGGTTGGTT
iNOS (NOS2)	TTCCAGAATCCCTGGACAAG	TGGGTCCTCTGGTCAAACTC
IL4	TCCTTACGGCAACAAGGAAC	GTGAGTTCAGACCGCTGACA
IL10	TAACTGCACCCACTTCCCAG	AGGCTTGGCAACCCAAGTAA
Arg1	ACAGACCGTGGGTTCTTCAC	TATCGGAGCGCCTTTCTCTA
SOCS3	CTTTACCACCGACGGAACCT	CCGTTGACAGTCTTCCGACA
CCL2	GATCCCAATGAGTCGGCTGG	ACAGAAGTGCTTGAGGTGGTT
