# Supplementary material for: Muscle stem cells and fibro-adipogenic progenitors in female pelvic floor muscle regeneration following birth injury
Source: NPJ Regen Med. 2022 Dec 16;7:72. doi: 10.1038/s41536-022-00264-1 (PMC9758192; doi:10.1038/s41536-022-00264-1)
Supplement: Supplementary file 2 — Reporting Summary [file 41536_2022_264_MOESM2_ESM.pdf]

nature portfolio

Corresponding author(s): Marianna Alperin
Last updated by author(s): 05/11/2022

Reporting Summary

Nature Portfolio wishes to improve the reproducibility of the work that we publish. This form provides structure for consistency and transparency in reporting. For further information on Nature Portfolio policies, see our Editorial Policies and the Editorial Policy Checklist.

Statistics

For all statistical analyses, confirm that the following items are present in the figure legend, table legend, main text, or Methods section.

n/a	Confirmed	
☐	☒	The exact sample size (*n*) for each experimental group/condition, given as a discrete number and unit of measurement
☒	☐	A statement on whether measurements were taken from distinct samples or whether the same sample was measured repeatedly
☐	☒	The statistical test(s) used AND whether they are one- or two-sided<br>*Only common tests should be described solely by name; describe more complex techniques in the Methods section.*
☒	☐	A description of all covariates tested
☐	☒	A description of any assumptions or corrections, such as tests of normality and adjustment for multiple comparisons
☐	☒	A full description of the statistical parameters including central tendency (e.g. means) or other basic estimates (e.g. regression coefficient) AND variation (e.g. standard deviation) or associated estimates of uncertainty (e.g. confidence intervals)
☐	☒	For null hypothesis testing, the test statistic (e.g. *F*, *t*, *r*) with confidence intervals, effect sizes, degrees of freedom and *P* value noted<br>*Give P values as exact values whenever suitable.*
☒	☐	For Bayesian analysis, information on the choice of priors and Markov chain Monte Carlo settings
☒	☐	For hierarchical and complex designs, identification of the appropriate level for tests and full reporting of outcomes
☒	☐	Estimates of effect sizes (e.g. Cohen's *d*, Pearson's *r*), indicating how they were calculated

Our web collection on statistics for biologists contains articles on many of the points above.

Software and code

Policy information about availability of computer code

Data collection	NA
Data analysis	Data were analyzed using GraphPad Prism v8.0, San Diego, CA.<br>Images were analyzed with ImageJ 1.51s, 1.53k, and Adobe Photoshop CS4 and 2022.

For manuscripts utilizing custom algorithms or software that are central to the research but not yet described in published literature, software must be made available to editors and reviewers. We strongly encourage code deposition in a community repository (e.g. GitHub). See the Nature Portfolio guidelines for submitting code & software for further information.

Data

Policy information about availability of data

All manuscripts must include a data availability statement. This statement should provide the following information, where applicable:

- Accession codes, unique identifiers, or web links for publicly available datasets
- A description of any restrictions on data availability
- For clinical datasets or third party data, please ensure that the statement adheres to our policy

All data generated or analysed during this study are included in this published article (and its supplementary information files).

Field-specific reporting

Please select the one below that is the best fit for your research. If you are not sure, read the appropriate sections before making your selection.

☒ Life sciences ☐ Behavioural & social sciences ☐ Ecological, evolutionary & environmental sciences

For a reference copy of the document with all sections, see nature.com/documents/nr-reporting-summary-flat.pdf

Life sciences study design

All studies must disclose on these points even when the disclosure is negative.

Sample size	A priory sample size calculation was not done given the novelty of the study. Our primary outcome fiber size at long term time point. Statistical significant difference between groups was observed in this primary outcome, eliminating concern for type 2 error.
Data exclusions	No data were excluded
Replication	1) All experiments had proper controls<br>2) All protocol used were previously tested and validated, and routinely used in the lab<br>3) None of the obtained results were excluded from the study<br>4) The investigators were blinded to group allocation during quantification and data analysis.<br>5) Animal were randomly allocated to study groups
Randomization	Animals were randomly allocated to the study groups
Blinding	For images quantification, we assigned numbers to each sample with no reference to the study group<br>For flow cytometry, the core that performed the sort did not know group allocation, thus the gaiting was only based on FMOs provided for the day.<br>For qPCR experiment, all samples were assigned a number and plates were ran based on those numbers.

Reporting for specific materials, systems and methods

We require information from authors about some types of materials, experimental systems and methods used in many studies. Here, indicate whether each material, system or method listed is relevant to your study. If you are not sure if a list item applies to your research, read the appropriate section before selecting a response.

Materials & experimental systems

n/a	Involved in the study	
☐	☒	Antibodies
☒	☐	Eukaryotic cell lines
☒	☐	Palaeontology and archaeology
☐	☒	Animals and other organisms
☒	☐	Human research participants
☒	☐	Clinical data
☒	☐	Dual use research of concern

Methods

n/a	Involved in the study	
☒	☐	ChIP-seq
☐	☒	Flow cytometry
☒	☐	MRI-based neuroimaging

Antibodies

Antibodies used	For IF: Primary antibodies used included: rabbit anti-laminin (Sigma, L9393, 1:200), mouse anti- eMyHC (Developmental Studies Hybridoma Bank (DSHB), F1.652, 1:200), mouse anti-Pax7 (Developmental Studies Hybridoma Bank (DSHB), Pax7-c, DSHB, 1:100), and rabbit anti-Ki67 (Abcam, ab15580, 1:100), goat anti-PDGFR-α (R&D Systems, AF1062, 1:100), α-SMA (Cell Signaling, 19245S, 1:100), collagen I (Invitrogen, PA5-95137, 1:200), CD45 (1:100), CD68 (Biorad, MCA341GA, 1:100), Perilipin (Fisher, PA1-1052, 1:200), synaptotagmin (Developmental Studies Hybridoma Bank (DSHB), znp-1, 1:500), bungarotoxin (Invitrogen, B13422, 1:250). Secondary antibodies included: Alexa Fluor 488 goat anti-mouse IgG (Invitrogen, A21121, 1:200 for eMyHC and 1:250 for Pax7), Alexa Fluor 546 goat anti-mouse IgG (Invitrogen, A11030, 1:250), Alexa Fluor 546 goat anti-rabbit IgG (Invitrogen, A11035, 1:500 for laminin and 1:250 for Ki67), Alexa Fluor 546 donkey anti-goat IgG (Invitrogen, A-11056, 1:250), Alexa Fluor 488 donkey anti-rabbit (Invitrogen, A-21206, 1:250).<br>For Flow: Anti CD45 (BD Biosciences, 565465, [0.3 ug/106 cells]), CD4 (Invitrogen, 11-0040-81, [0.2 ug/106 cells]), and CD8 (Biolegend, 200608, [0.2 ug/106 cells]).
Validation	For IF: All antibodies were tested in tissues where the protein of interest was known to be expressed. We also employed negative controls to test for not specific binding.<br>For flow: CD45 antibody was previously titrated and validated (Boscolo Sesillo et al. 2020). CD4 and CD8 antibodies were tested and titrated before starting the sorting experiment for this study. Six different amounts were used for titration for CD4 (0, 0.15, 0.2, 0.25, 0.3, 0.35 ug) and CD8 (0, 0.1, 0.15, 0.2, 0.25, 0.3 ug) antibodies. Titration data can be provided if needed.

Animals and other organisms

Policy information about studies involving animals; ARRIVE guidelines recommended for reporting animal research

Laboratory animals	Female 3-month old Sprague-Dawley rats (Envigo, Indianapolis, IN).
Wild animals	*Provide details on animals observed in or captured in the field; report species, sex and age where possible. Describe how animals were caught and transported and what happened to captive animals after the study (if killed, explain why and describe method; if released, say where and when) OR state that the study did not involve wild animals.*
Field-collected samples	*For laboratory work with field-collected samples, describe all relevant parameters such as housing, maintenance, temperature, photoperiod and end-of-experiment protocol OR state that the study did not involve samples collected from the field.*
Ethics oversight	*Identify the organization(s) that approved or provided guidance on the study protocol, OR state that no ethical approval or guidance was required and explain why not.*

Note that full information on the approval of the study protocol must also be provided in the manuscript.

Flow Cytometry

Plots

Confirm that:

☒ The axis labels state the marker and fluorochrome used (e.g. CD4-FITC).

☒ The axis scales are clearly visible. Include numbers along axes only for bottom left plot of group (a 'group' is an analysis of identical markers).

☒ All plots are contour plots with outliers or pseudocolor plots.

☒ A numerical value for number of cells or percentage (with statistics) is provided.

Methodology

Sample preparation	Cell isolation was performed using Ham's F-10 supplemented with 10% horse serum media. TA, C, ICa, and PCa were individually minced and incubated in 700 units/ml collagenase type II solution for 1.5 hours and collagenase and dispase II solution (100 units/mL and 2 units/mL, respectively) for 30 minutes. Tissue was then passed through a 20G needle and a 70 µm nylon filter. Antibody incubation was performed in 1 mL volume for 1 hour. Anti CD45 (BD Biosciences, 565465, [0.3 ug/106 cells]), CD4 (Invitrogen, 11-0040-81, [0.2 ug/106 cells]), and CD8 (Biolegend, 200608, [0.2 ug/106 cells]) antibodies were used to identify general immune cells population, T-helper cells, cytotoxic T-cells, respectively. All antibodies were first tested and titrated to determine the proper concentration to use for cell isolation.
Instrument	Cell Sorters: FACSAria II and FACSAria Fusion (BD Biosciences, USA)<br>Analyzer: BD LSRFortessa x20
Software	FlowJo
Cell population abundance	CD45:15-25%; CD45+/CD4-/CD8-: 1-5%; CD4: 1-2%; CD8: 0.5-1.5% Over the total live cells
Gating strategy	Remove debris; selected for single cells; selected live cells; selected CD45+ population; selected CD8+, CD4+, and CD45+/ CD4-/CD8- were sorted

☒ Tick this box to confirm that a figure exemplifying the gating strategy is provided in the Supplementary Information.
